# Chemical diplomacy in male tilapia: urinary signal increases sex hormone and decreases aggression

**DOI:** 10.1038/s41598-017-07558-1

**Published:** 2017-08-09

**Authors:** João L. Saraiva, Tina Keller-Costa, Peter C. Hubbard, Ana Rato, Adelino V. M. Canário

**Affiliations:** Centre of Marine Sciences (CCMAR), University of Algarve, Campus de Gambelas, 8005-139 Faro Portugal

## Abstract

Androgens, namely 11-ketotestosterone (11KT), have a central role in male fish reproductive physiology and are thought to be involved in both aggression and social signalling. Aggressive encounters occur frequently in social species, and fights may cause energy depletion, injury and loss of social status. Signalling for social dominance and fighting ability in an agonistic context can minimize these costs. Here, we test the hypothesis of a ‘chemical diplomacy’ mechanism through urinary signals that avoids aggression and evokes an androgen response in receiver males of Mozambique tilapia (*Oreochromis mossambicus*). We show a decoupling between aggression and the androgen response; males fighting their mirror image experience an unresolved interaction and a severe drop in urinary 11KT. However, if concurrently exposed to dominant male urine, aggression drops but urinary 11KT levels remain high. Furthermore, 11KT increases in males exposed to dominant male urine in the absence of a visual stimulus. The use of a urinary signal to lower aggression may be an adaptive mechanism to resolve disputes and avoid the costs of fighting. As dominance is linked to nest building and mating with females, the 11KT response of subordinate males suggests chemical eavesdropping, possibly in preparation for parasitic fertilizations.

## Introduction

Androgens, synthesized mainly in the gonads and adrenal tissue^1^, are essential in vertebrate reproductive physiology and behaviour^2^. In teleost fishes, the main androgen 11-ketotestosterone (11KT) is associated with male reproductive potential^3, 4^, promotion of spermatogenesis and spermiation^5^, development of secondary sex characters^6–8^, and expression of reproductive behaviours^7, 9, 10^.

The mechanisms underpinning male aggression have also been suggested to be associated with androgens^2, 11–14^, which are responsive to social challenges^15, 16^; circulating levels usually rise in winners^17, 18^, increasing the probability of winning ensuing fights and the willingness to engage in further disputes^17, 19^, and they may fall in losers^20^. The simple anticipation of an agonistic encounter is enough to evoke an acute rise in circulating 11KT^21^. However, the relationship between social behaviour and androgens is not completely clear; for example, castrated Mozambique tilapia (*Oreochromis mossambicus*) males cease reproductive displays but continue to exhibit aggressive behaviour^9^, and mirror-induced fights do not evoke a rise in androgens^22^. The endocrine response to social challenges may therefore be dependent on the appraisal of the situation^23^, and rely on cues conveying likely conflict outcome or relative fighting ability, rather than fighting *per se* ^24^.

Social animals use aggression to increase access to food, territories, mates and other resources, and to ascend in the social hierarchy e.g. refs 25–28. In males, social dominance stimulates spermatogenesis, whereas subordination generally inhibits the reproductive axis^29^, besides inflicting social costs^20^. The outcome of a contest may therefore have consequences beyond the original dispute; while the costs of fighting may be offset by the benefits of victory, losing can impair the chances of reproduction and survival. Signalling dominance deters fights and circumvents these costs with obvious advantages: faster conflict resolution; less energy expenditure; and lower risk of injury^30, 31^. In social species where dominance signals are reliable predictors of conflict outcome, both winners and losers benefit from ritualized interactions. Both may use signals to convey fighting ability and motivation; the individual signalling higher dominance wins more often, and without recourse to violence^32–37^. This falls under the general theoretical framework for the evolution of animal signals^38, 39^, in which the information conveyed alters the behaviour of the receiver and works in favour of the signaller^39^. There are numerous examples of the benefits of signallers communicating dominance^40^. However, there are far fewer examples of benefits for the receivers, as these solely occur if both signaller and receiver place the predicted outcome of the interaction in the same order of preference^41^ or if they both have a common interest, such as avoiding an escalated fight^39^. In fish, signals and cues can be visual^42^, acoustic^43^, chemical^44^ or multimodal^45^.

Chemical signals have been shown to stimulate androgen secretion in males of several fish species. In male goldfish *Carassius auratus*, for example, both female and male chemical signals induce changes in physiology^46, 47^. Particularly, the reproductive system of receiver males is stimulated upon smelling other sexually mature males’ odours^48^. Male goldfish adaptively increase milt production in advance of an approaching ovulation by monitoring female condition both directly – by detecting the pre-ovulatory steroid pheromone – and indirectly – by detecting cues which the pre-ovulatory female has stimulated other males to release^47^. Male mangrove rivulus *Kryptolebias marmoratus* also increase androgen levels when smelling chemical signals from conspecific males^49^.

Here, we focus on the chemical dominance signal in the Mozambique tilapia and its effect on receiver males. This species is a lek-breeding, mouth-brooding African cichlid. Males form spawning aggregations (leks) where they dig depressions in the substrate and display to females. Males fight for the best position (usually near the centre), and a social hierarchy ensues^50, 51^. Dominant males use chemical communication and strategically emit pulses of urine in the presence of females and rival males^33–35^. Thereafter, the hierarchy is stabilized and disputes are resolved with less violence. In cases where the males are either unable to smell or to release urine, the conflict rapidly escalates to violent attacks resulting in an unstable social scenario^33^. In a mirror test, when males are simultaneously stimulated with dominant male urine (DMU), aggression decreases markedly, suggesting the presence of an appeasing chemical cue in the urine^52^. Other cichlids have also been shown to signal dominance chemically through faeces^53^ and to mediate aggressive interactions in male-male encounters through the emission of urine pulses^54^.

In this paper, we aim to answer the question: do chemical signals play a role in the aggressive and endocrine response of male Mozambique tilapia in agonistic encounters? To answer this, we analyse behaviour and hormones of male Mozambique tilapia responding to a chemical signal from dominant males, using two approaches: 1) a simulated territorial intrusion using a mirror test, and 2) an olfactory stimulation test with DMU without any visual cue.

In the mirror test, we presented the focal males with their mirror image simulating a territorial intrusion, which is known to trigger aggression in an unresolved dispute^22^. In the experimental group we provided pulses of DMU to the focal male as an olfactory cue suggesting the dominance status of the intruder (the mirror image), while in the control group, the stimulus was their own tank-water. We analysed aggressive behaviour and the change (Δ) in urinary steroids (11KT and cortisol) as the difference in levels 24 hours before and immediately after the 20 min exposure to the mirror. Using this established measure allows to control for initial differences in hormone levels among test subjects^22^. While there is no hormonal response in tied fights against a mirror^22^, we hypothesised that DMU would not only reduce aggression but also prompt 11KT to drop even in the absence of a visual stimulus; the male would interpret the olfactory cue as the presence of dominant male(s), which would cause an endocrine ‘loser effect’ and 11KT suppression. In contrast, the alternative hypothesis was that 11KT may rise even if aggression decreases. This could be explained by associative inference; the receiver (focal) male would associate the presence of a dominant male(s) with the vicinity of spawning females in the lek, thus stimulating the reproductive axis of the receiver.

In the olfactory stimulation test, we assessed the endocrine effect of smelling DMU by measuring the release rate of 11KT (measured non-invasively in the holding water) in isolated males (with no visual stimulus) before and after stimulation. We expected hormone levels to follow the same pattern as in the mirror experiment, confirming chemical signals as an essential component of social information, even in the absence of visual or auditory stimuli. Although our main focus was on 11KT, cortisol was also measured to assess the acute stress effect of smelling DMU.

## Results

### Mirror test

#### Behaviour

**Figure 1:**
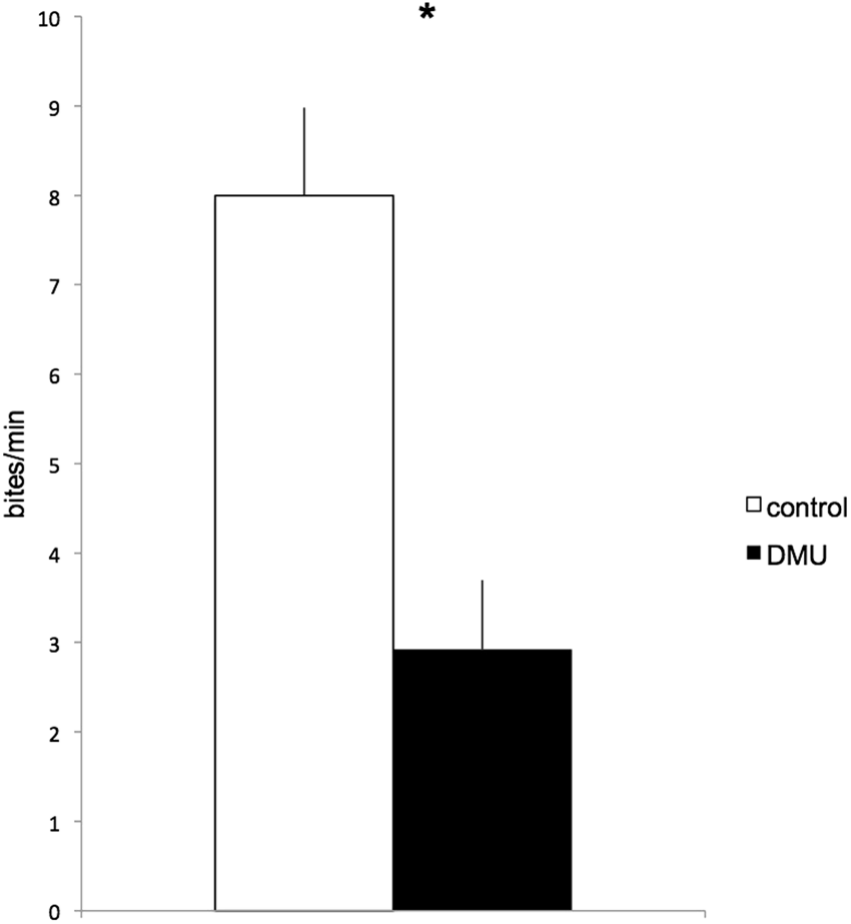
Frequency of bites per minute against the mirror in control (white bar) and DMU stimulated males (black bar). N_DMU_ = 22, N_control_ = 11 Significant differences at *P* < 0.05 are marked with *. See text for details.

Males stimulated with DMU were significantly less aggressive towards their mirror image than controls (DMU = 2.92 ± 0.86 *bites*.min^−1^, Control = 8.00 ± 0.98 *bites*.min^−1^, N_DMU_ = 22, N_control_ = 11, Student’s t = 3.62, *P* = 0.001, Fig. 1).

#### Hormones

Prior to the mirror test, there were no differences in the initial levels of urinary cortisol and 11KT between fish stimulated with DMU or control water (cortisol: DMU = 15.86 ± 1.43 ng.ml^−1^, Control = 16.19 ± 2.16 ng.ml^−1^, N_DMU_ = 13, N_control_ = 6, Student’s t = 0.13, *P* = 0.897; 11KT: DMU = 28.79 ± 3.55 ng.ml^−1^, Control = 37.17 ± 4.07 ng.ml^−1^, N_DMU_ = 13, N_control_ = 6, Student’s t = 1.4, *P* = 0.177).

**Figure 2:**
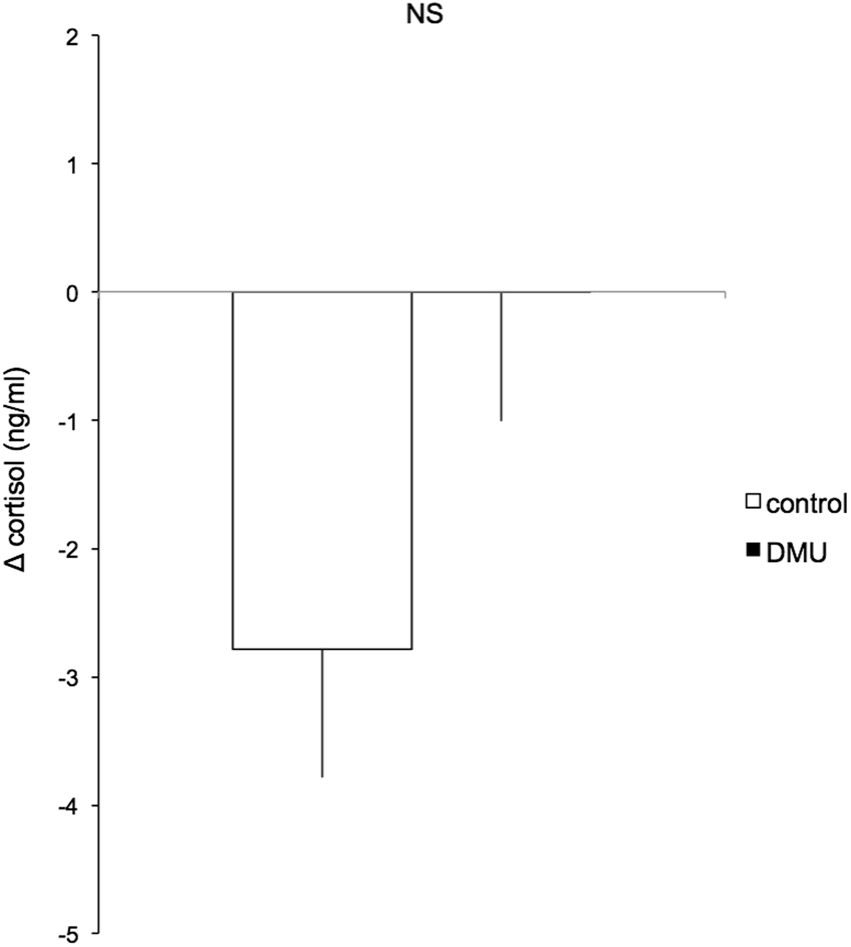
Changes (Δ) in urinary cortisol levels (as measured immediately after the mirror test and subtracted by the urinary cortisol levels measured 24 h before) in control (white bar) and DMU stimulated males (black bar). N_DMU_ = 13, N_control_ = 6. No significant difference was found between treatments (NS). See text for details.

**Figure 3:**
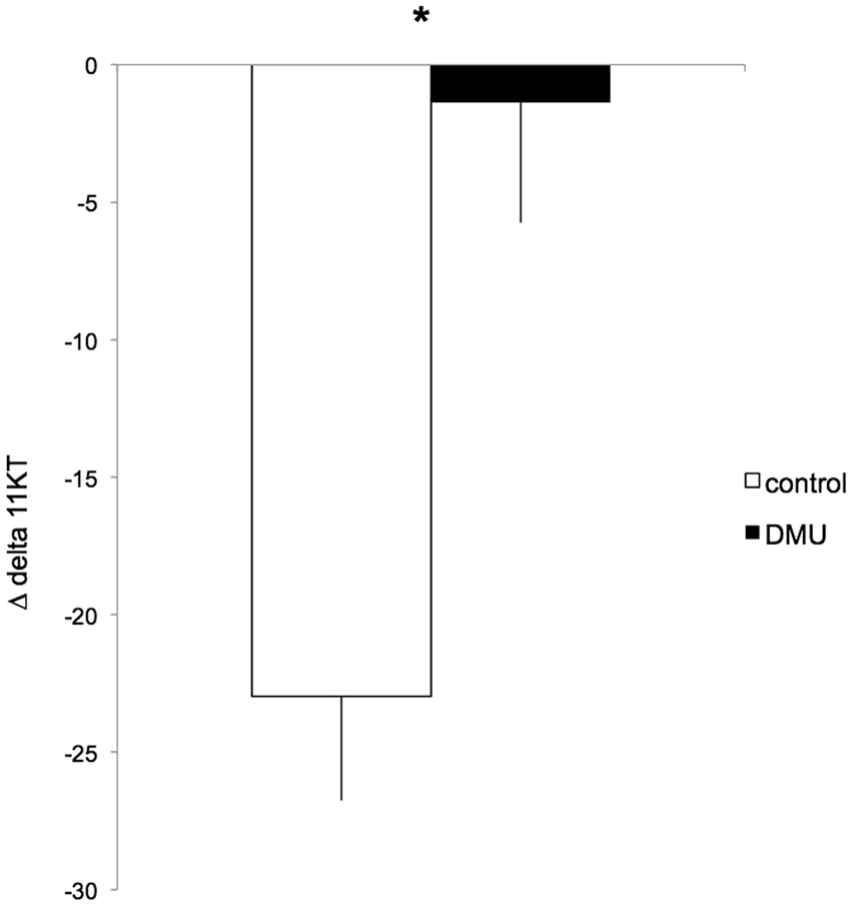
Changes (Δ) in urinary 11KT levels (as measured immediately after the mirror test and subtracted by the urinary 11KT levels measured 24 h before) in control (white bar) and DMU (black bar) stimulated males. N_DMU_ = 13, N_control_ = 6. Significant differences at *P* < 0.05 are marked with *. See text for details.

The change in urinary cortisol as a result of the mirror test, did not differ significantly between DMU and control males (Δ-cortisol: DMU = −0.01 ± 1.53 ng.ml^−1^, Control = −2.78 ± 1.28 ng.ml^−1^, N_DMU_ = 13, N_control_ = 6, Student’s t = 1.14, *P* = 0.271, Fig. 2). However, the drop in urinary 11KT was significantly greater in the control group than in the DMU group (Δ−11KT DMU = −1.37 ± 4.38 ng.ml^−1^, Control = −22.98 ± 3.78 ng.ml^−1^, N_DMU_ = 13, N_control_ = 6, Student’s t = 3.09, *P* = 0.007, Fig. 3).

Final cortisol levels did not differ in any of the groups (cortisol DMU_before_ = 15.86 + 1.43 ng.ml−1, DMU_after_ = 15.85 ± 1.85 ng.ml−1, N = 13, paired Student’s t = 0.006, P = 0.995; cortisol Control_before_ = 16.19 ± 2.16 ng.ml−1, Control_after_ = 13.41 ± 2.06 ng.ml^−1^, N = 6, paired Student’s t = 2.181, P = 0.081). Interestingly, while no significant change in urinary 11KT was seen in males stimulated with DMU, the final urinary levels of 11KT were significantly lower than the initial levels of control fish (11KT DMU_before_ = 28.79 ± 3.55 ng.ml^−1^, DMU_after_ = 25.04 ± 4.21 ng.ml^−1^, N = 13, paired Student’s t = 0.90, *P* = 0.386; 11KT Control_before_ = 37.17 ± 4.07 ng.ml^−1^, Control_after_ = 14.19 ± 2.50 ng.ml^−1^, N = 6, paired Student’s t = 6.08, *P* = 0.002).

#### Olfactory stimulation test

**Figure 4:**
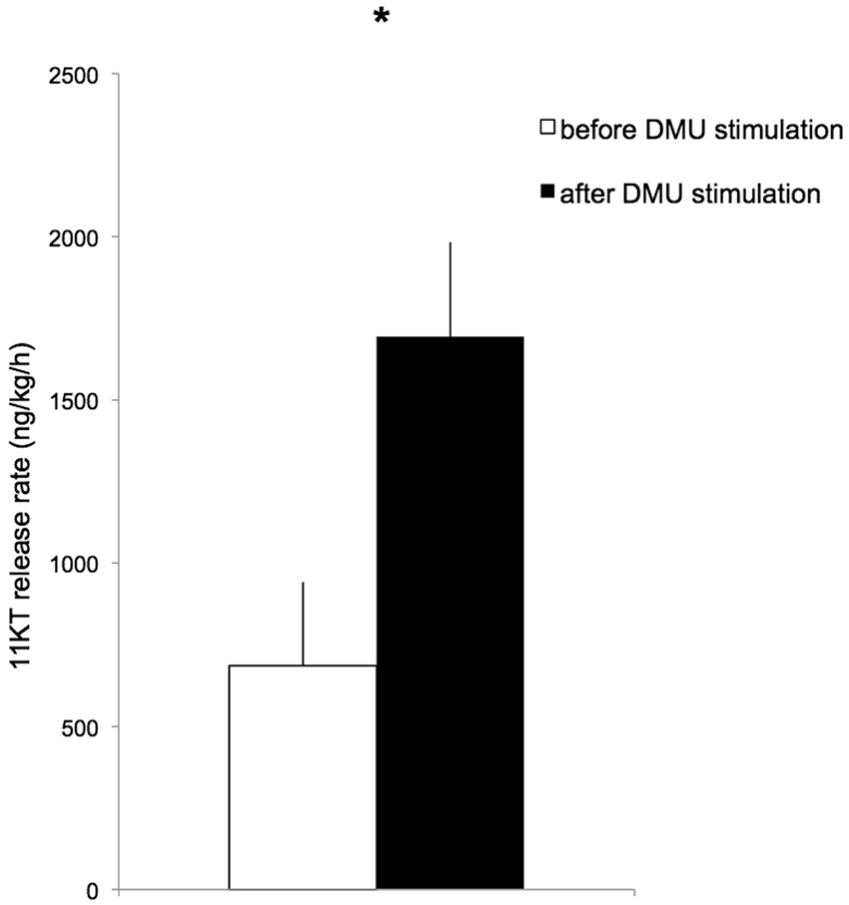
Release rate of 11KT into the tank holding water of males before (white bar) and after (black bar) stimulation with DMU without any visual stimulus from another male (or mirror image). N = 8. Significant differences at *P* < 0.05 are marked with *. See results for details.

The 11KT release rate increased 3-fold in isolated males exposed to DMU (release rate before stimulation = 686.39 ± 255.81 ng.h^−1^.kg^−1^, release rate after stimulation = 1694.45 ± 289.27 ng.h^−1^.kg^−1^, N = 8, paired Student’s t = 6.034, *P* = 0.001 (Fig. 4).

## Discussion

Signalling of social rank in fishes is likely to be achieved through a combination of signals: colouration changes^55, 56^ and sounds^43, 57^, for example, are important elements in fish communication. Our results show, however, that not only are chemical signals alone enough to elicit an endocrine response in males, they seem to be effective even in the presence of a relevant visual stimulus. The data presented here suggest that DMU contains a chemical signal that decreases aggression and stimulates 11KT secretion. These effects cannot be attributed to stress, since smelling DMU does not alter cortisol levels and therefore the response seems to be specific to the hypothalamus - pituitary - gonad (HPG) axis.

While control males fighting the mirror experience a drop in 11KT, probably due to the lack of social stimuli during the social isolation period prior to the experiment^58^, males simultaneously exposed to DMU show elevated levels of this androgen. Our explanation is that chemical information about the likely outcome of the fight is contained in DMU, since the physiological response to agonistic encounters depends on the cognitive appraisal that the individuals make of the situation^23, 24^. This seems to confirm our alternative hypothesis: males smelling DMU maintain high levels of 11KT because they do not perceive the result of the interaction as a defeat, despite the reduction of their aggression. The adaptive benefits of reducing attacks in the presence of chemical information about the opponent’s relative fighting ability include energetic savings and fitness maintenance. In addition, decreasing aggression in dyadic fights with no audience may not represent a real threat to social status because of the missing ‘audience effect’^20^. Examples of audience effect include raised hormone levels in male Mozambique tilapia as a result of watching fights^59^, the frequency shifts of male aggressive displays in Siamese fighting (*Betta splendens*) fish as a function of the sex of the audience^60^ and the direct influence on the chances of future success in the Japanese quail^20^.

In this context, chemical signals of dominance may play a diplomatic role, promoting rapid conflict resolution while still enabling future chances for reproductive success in the subordinate. This chemical diplomacy is adaptive for the dominant (who wins the interaction and accesses the disputed resource without fighting) and for the subordinate (who loses the interaction but, nevertheless, does not experience the loser effect). On a social perspective, lower-rank males use the chemical information on the relative fighting ability of their opponent to avoid the costs of fighting. Such a mechanism works in the interest of both the subordinate and the dominant and may thus be a stable strategy^39^. Furthermore, the dominance hierarchies are dynamic; a subordinate at one time may become a dominant at another. The chemical diplomacy mechanism and its adaptive value can thus be carried across social ranks. An interesting topic for future research would be to determine the speed at which this dominance signal is modified as the male moves up or down the social ladder.

Apart from the agonistic perspective, the scent of a dominant male also seems to stimulate the subordinate’s reproductive system, as 11KT is involved in reproductive potential^3–5^. While apparently paradoxical, the explanation for this effect may lay in the lek breeding system of this species. Dominant males attract and prime mature females using a reproductive pheromone in the urine^61, 62^. Therefore, in a lek arena there is a high probability of finding females in the vicinity of a urinating dominant male. Receivers may thus be stimulated by the chemical signals present in dominant male urine and mount a physiological response in order to take advantage of the situation and try to mate, either in a neighbouring nest or attempting a parasitic fertilization^63^- a possible case of chemical eavesdropping. Similarly, mangrove rivulus *Kryptolebias marmoratus* males experience a rise in androgens when stimulated chemically (but not visually) with water from conspecific males^49^.

In the present study, we consider mirror fighting to be a valid proxy to study intra-specific aggression and chemical communication (see Methods: the mirror test). This species relies not only on lateral and frontal displays (low level or restrained aggression) but also on frontal bouts and mouth fights (high level or overt aggression). With the exception of circular displays (which are not possible against a mirror), the whole range of aggressive behaviours occurs in the mirror experiments culminating in mouth fights, similar to natural dyadic agonistic encounters^22, 52, 63, 64^. In addition, the mirror assay allows control over the chemical cues received by the focal male, which would be far more difficult using other experimental approaches.

Our results show a decoupling between androgens and aggression. Control males fought the mirror intensely, yet their 11KT levels decreased; conversely, males smelling DMU reduced their aggression but their 11KT remained high. This supports the observations with castrated tilapia, which continued to show aggressive behaviour despite having only residual circulating 11KT^9^. It is possible that two neuroendocrine pathways for DMU signalling are present in male tilapia; one that involves brain regions controlling aggression (the ‘social brain network’), and the other that stimulates 11KT secretion, probably via the HPG axis. It remains to be determined whether these dual pathways are activated by the same chemical signal(s) in the urine, and whether the same steroidal pheromone that primes the female reproductive system does so in males^35, 61, 62^, since it has no clear effect on suppression of aggression *per se* ^52^.

In conclusion, we show that chemical signals in DMU evoke an adaptive, diplomatic, conflict resolution response that reduces aggression without depressing the endocrine system of the receiver. These actions may involve two neural pathways; one directed at aggression brain centres, and the other directed at the HPG axis, which stimulates androgen production. This will be the subject of future research.

## Methods

### Ethics statement

All fish used in this experiment belong to a brood stock maintained at the Algarve Centre of Marine Sciences (CCMAR). CCMAR facilities and their staff are certified to house and conduct experiments with live animals (‘group-1’ license by the ‘Direção Geral de Veterinária’, Ministry of Agriculture, Rural Development and Fisheries of Portugal). The internal ethics committee at CCMAR approved the experimental procedures. This study was carried out according to the ASAB guidelines for the use of animals in research^65^.

### Experimental animals

Tilapia were reared in 500 L stock tanks equipped with sand substratum in dechlorinated, aerated freshwater and under constant temperature (26 °C) and photoperiod (12 h light:12 h dark) conditions. Fish were fed once a day with commercial cichlid food pellets. The fish were anaesthetized with a 1:1 mixture of MS-222 (3-aminobenzoic acid ethyl ester; Sigma-Aldrich) and sodium bicarbonate for morphometric measurements (i.e. standard length SL in mm and body weight BW in g) and tagging (T-Bar anchor FD94, Floy Tag Inc., Seattle, WA, USA) to allow individual identification.

### Assessment of male social status and urine collection

In total, twenty-five social groups were formed, each with five size-matched males and five females and housed in 250 L aquaria with sandy substrate. Every male in each group was focally observed for five min per day over five consecutive days. We registered the frequency of agonistic interactions: chases, bites, mouth-to-mouth fights and submission. The outcomes of these interactions (victories versus defeats) were used to calculate a dominance index (DI: number of victories/total number of interactions) ranging from 0 to 1 for each male throughout the observation period (see refs 33, 62 for more details). After each daily observation, all males had their urine collected by gently squeezing the abdominal area above and anterior to the urogenital papilla. All urine samples were immediately stored at −20 °C until use in the mirror tests.

Only urine from dominant males (i.e. that showed a DI consistently higher than 0.5 throughout the 5 days of observation) was used for the tests, with an equal contribution of 1 ml of urine from each male. This resulted in a 30 ml pool of dominant male urine (DMU) collected from 30 males. This was introduced as a chemical stimulus for the males interacting with their own mirror image. Donor males had a mean DI of 0.78 ± 0.03 and a mean body weight of 81.8 ± 7.39 g (mean ± SEM). Non-donor males had a mean DI of 0.31 ± 0.04 and a mean body weight of 76.5 ± 4.56 g.

The use of pooled samples has methodological justifications; it would have been impossible to collect sufficient urine from one individual dominant male to conduct the whole experiment. The largest dominants exceptionally provide a maximum of 1.5 ml of urine per extraction; in order to perform the experiment we needed a much larger volume, and this could only be achieved by pooling. As yet, there is no evidence that tilapia perceive a urine pool as coming from several, or one individual. The alternative would to use in each trial a different urine sample from a different donor individual, but that would create a confounding effect and likely adds additional variation to the data due to variations in the potencies of individual male urine samples. We therefore decided to standardize the stimulus by pooling the urine of these 30 selected dominant males.

### The mirror test

The use of mirror assays in studies of aggressive behaviour has been a motive for debate^66, 67^. For example, one study^68^ found that male fish (*Haplochromis burtoni*) fighting their mirror image experience different brain responses compared to males fighting a real opponent, while the males’ hormonal responses (as measured in the blood plasma) were found to be similar in the two experimental set-ups. However, a commentary on this study has argued that these findings could be biased by several factors: extremely low hormone concentrations atypical for the taxon; blood plasma hormones *versus* hormones measured in urine or from droppings; discrepancies in behavioural tasks that hinder a correct comparison between the mirror and a real opponent^69^. Therefore, despite some controversy around mirror tests we sought to use it as a simple way to manipulate chemical stimulation while keeping other factors under control.

Males used in the mirror test were a separate, unfamiliar set from the DMU donors and were taken randomly from stock tanks. They underwent a gradual process of social isolation to standardize short-term social experience and hormone levels: they were housed for seven days with four females and no other male, and transferred to the experimental tank where they remained alone for seven more days. This tank held a partition concealing a mirror. A tube was fixed near to the mirror, through which the stimulus (DMU or water) was delivered. All males tested built nests in their experimental tanks during this isolation period. On day seven, the partition concealing the mirror was lifted. A similar set up and procedure has been used previously^22^. Males that showed no reaction towards the mirror for 20 min did not receive a stimulus and where excluded from the experiment. As a result, 22 males were considered valid in the DMU group and 11 in the control. Of these, only 13 males provided urine before and after the test in the DMU group, while 6 did so in the control, allowing the calculation of the difference in hormone concentration of urinary 11KT and cortisol between the two time points. The mean body weight of males in the DMU group was 43.1 ± 5.6 g and in the control group was 39.5 ± 3.2 g (mean ± SEM). Body weights in both groups did not differ statistically (Student’s *t*-test t = 0.55, *P* = 0.59).

Stimulus donors were larger than test males (DMU donors vs DMU receivers: 81.8 ± 7.39 g vs 43.1 ± 5.6 g, Student’s *t*-test t = −2.53, *P* = 0.016; DMU donors vs control: 81.8 ± 7.39 g vs 39.5 ± 3.2 g, Student’s *t*-test t = −3.27, *P* = 0.002). However, previous studies have demonstrated that the olfactory potency of male tilapia urine is correlated with male social rank rather than the body size of the males^34^.

Immediately before the start of a behavioural trial, a 1 ml aliquot of the urine pool was thawed and diluted 1:100 v/v in water collected from the recirculating assay system at an outlet after the filter passage. Tilapia have high olfactory sensitivity to DMU, even in at very low concentrations (see refs 35 and 34 for details on olfactory sensitivity and concentrations). The pool and dilution factor we used have been tested and validated in ref. 52. For males reacting to the mirror image, immediately upon the initial response a peristaltic pump was turned on and the chemical stimulus (DMU or control water) was delivered in pulses and at a flow rate of 20 ml/min, at five intervals of 1 min flow-on followed by 1 min flow-off, covering a total of 10 min of stimulus delivery. This was followed by another 5 min in which males were allowed to continue their interaction with the mirror, resulting in a total observation period of 15 min per male. Male behaviour was recorded on video using a remote controlled video camera so that the animals were not disturbed by the observers. The recorded behaviour was analysed using the Observer XT software (Noldus, The Netherlands). See ref. 52 for a detailed explanation of the experimental set-up.

Urine was sampled from the receiver males 24 h before the mirror test and immediately after the test, and stored at −20 °C for further analysis.

All the assays were balanced and randomized, i.e., in all mirror trials we used controls and DMU treatments, and the sequence in which the fish were tested was randomized to control for order effects.

### The olfactory stimulation test

We used a non-invasive method to test the endocrine effect of DMU on steroid release on a different set of mature males. The DMU used as stimulus in this olfactory stimulation test belonged to a pool of urine different from the one used in the mirror assay. In this case, urine was collected from 8 donor males ca 100 g, housed each with 4 females for at least 2 weeks. Electro-olfactogram recordings confirmed that the olfactory potency of urine from males reared solely with females and dominants from a mixed sex group are similar.

Each test male was randomly taken from the stock tank and placed in a glass aquarium with 6 L de-chlorinated freshwater at 27 °C (±1 °C), equipped with an air supply, where individuals were isolated and maintained overnight. The next day, the fish were transferred to an identical aquarium with a volume of clean de-chlorinated water normalized to the body weight of the fish (1 L of water per 10 g of fish according to refs 61, 70). One hour after the transfer, 1 L of water was collected (control sample, at time 0 h), through siphoning with a tube previously placed in the tank. After collection of the first water sample, the collected volume was replaced with clean de-chlorinated freshwater and the chemical stimulus (DMU) was added to the aquarium to obtain a final dilution of 1:10 000 (e.g. to a 100 g male in 10 L of water, 1 ml of DMU was added). One hour later, another water sample (1 L) was collected (treatment sample, at time +1 h), using the same sampling procedure as described above. To avoid overestimations of the 11KT concentrations in the +1 h water samples due to 11KT present in the DMU stimulus, we also quantified the 11KT concentration in the DMU stimulus and subtracted this value from the final 11KT concentration in the holding water.

All male holding water samples were immediately C18-solid-phase extracted (C18-SPE) to concentrate the less polar compounds (including steroids) present in the water samples collected before and after the exposure to DMU. The 500 mg C18-SPE cartridges (‘Isolute’; International Sorbent Technology Ltd., Hengoed, UK) were activated with 5 ml of methanol (Sigma- Aldrich) followed by a washing step with 5 ml of distilled water. Each sample of male holding-water was passed through a separate C18-SPE cartridge using a vacuum pump (max. flow rate 1 ml/min). Subsequently, the compounds retained on each C18-SPE cartridge were eluted with 5 ml of methanol. The eluate (containing steroids) was immediately stored in glass vials at −20 °C until being analysed by radio-immunoassays (see below).

### Hormone levels and steroid radioimmunoassays (RIAs)

All hormone levels refer to the sum of free and conjugated steroids (steroid sulphates and glucoronates). The changes in urinary hormone concentrations (Δ) were calculated per individual as: (urine levels of hormone immediately after the test) - (urine levels of hormone 24 h before the test). RIAs were conducted following the protocol described in ref. 71. The antibody used for the 11KT assay was kindly donated by D. E. Kime and the cross-reactivity has been described in ref. 72. The cross-reactivity for cortisol was described in ref. 73.

### Statistics

The software SPSS 20.0 was used for all statistical procedures. To test for differences in aggression, hormone levels and the Δ in hormone levels between DMU- and control stimulated males in the mirror test we used Student’s *t* tests. To test for differences in release rates of 11KT in the olfactory stimulation test over time we used paired Student’s *t*-tests. Test values are provided in the results section.
